# Strategic Preparedness of Broad‐Spectrum Antivirals for Rapid Response Towards Next Pandemics

**DOI:** 10.1002/smsc.202500480

**Published:** 2026-01-14

**Authors:** Sanoj Rejinold N, Geun‐woo Jin, Jin‐Ho Choy

**Affiliations:** ^1^ Intelligent Nanohybrid Materials Laboratory (INML) Department of Chemistry College of Science and Technology Dankook University Cheonan 31116 Republic of Korea; ^2^ R&D Center Hyundai Bioscience Co. LTD. Seoul 07990 Republic of Korea; ^3^ Division of Natural Sciences The National Academy of Sciences Seoul 06579 Republic of Korea

**Keywords:** broad‐spectrum antivirals, drug repurposing, nanoengineered niclosamide, pandemic preparedness, targeted antiviral therapy

## Abstract

The COVID‐19 pandemic has underscored the urgent need for broad‐spectrum antivirals (BSAs) capable of countering diverse and rapidly emerging viral threats. Unlike virus‐specific drugs, BSAs offer cross‐family protection and can serve as adaptable therapeutic platforms for pandemic preparedness. Advances in nanotechnology have further strengthened this approach by improving the solubility, stability, and targeted delivery of antiviral agents. Several repurposed drugs, such as niclosamide, favipiravir, remdesivir, nitazoxanide, and zinc‐ionophores, have demonstrated potential broad‐spectrum activity when formulated at the nanoscale. These nanoengineered platforms enhance pharmacokinetic performance, tissue penetration, and bioavailability, thereby enabling lower effective doses and reduced systemic toxicity. Such nanotechnological strategies not only improve antiviral efficacy across multiple viral families, including Coronaviridae, Flaviviridae, Orthomyxoviridae, and Poxviridae, but also support scalable, cost‐effective production suitable for global deployment. By integrating drug repurposing with nanoengineering, BSAs can form the cornerstone of future pandemic preparedness, bridging the gap between laboratory innovation and rapid clinical response to emerging infectious diseases.

## Graphical abstract

Nanoengineered broad‐spectrum antivirals (BSAs) represent a transformative approach to pandemic preparedness. Unlike virus‐specific drugs requiring separate development, BSAs act across multiple viral families through nanoengineering strategies that enhance solubility, bioavailability, and host‐targeted activity. This review highlights niclosamide‐based nanohybrids and related platforms as adaptable, rapid‐response therapeutics for future viral outbreaks and global health resilience.

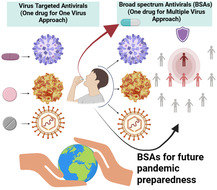

## 1 Introduction

The emergence of SARS‐CoV‐2 in late 2019 triggered a global health emergency that quickly escalated into a pandemic, overwhelming healthcare systems worldwide.^[^ ^1^ ^]^ Even years later, the virus continues to evolve in 2023, after China lifted strict containment policies, highly transmissible Omicron subvariants caused major surges, with hundreds of millions projected to be infected.^[^ ^2^ ^]^ Despite rapid drug repurposing efforts, the absence of broad‐spectrum antivirals (BSAs) severely limited our ability to respond effectively.^[^ ^3^ ^]^ This deficiency has since been recognized as a key vulnerability in pandemic preparedness.^[^ ^4^ ^]^ Today, emerging threats such as the panzootic spread of H5N1, with recent mammalian and cattle infections in the USA and recurrent outbreaks of Nipah virus in South Asia with high fatality rates highlight the continued urgency.^[^ ^5^, ^6^ ^]^ Additionally, MERS‐CoV remains endemic with a ≈35% case‐fatality rate.^[^ ^7^ ^]^ These developments reinforce the critical global need to develop BSAs capable of responding swiftly to future viral pandemics.

**Figure 1:**
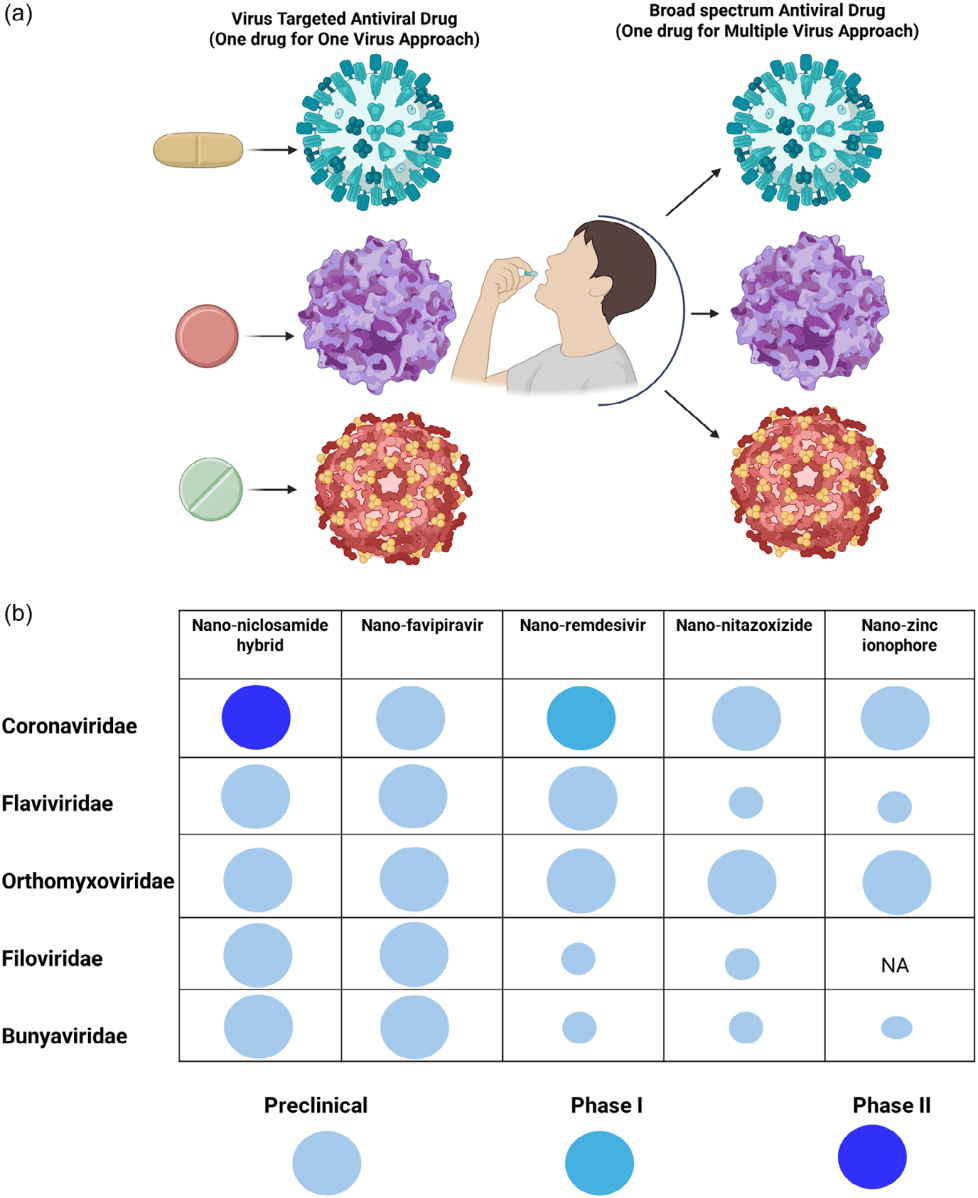
Comparative and translational mapping of BSA strategies and their nanoengineered platforms. a) Schematic comparison between virus‐specific and BSA paradigms. The left panel illustrates the “one‐drug‐one‐virus” approach, in which each antiviral molecule targets a single pathogen, requiring separate drug development for every emerging viral threat. In contrast, the right panel represents the “one‐drug‐many‐viruses” concept of BSAs, where a single therapeutic—often nanoengineered for enhanced solubility, stability, and targeted delivery—acts against multiple viral families, enabling rapid therapeutic deployment during outbreaks. b) Data‐driven bubble‐matrix visualization linking representative nanoengineered BSAs (nano‐NIC hybrid (NIC–MgO–HPMC), nano‐favipiravir, nano‐remdesivir, nano‐NTZ, and nano‐zinc‐ionophore) to their principal viral‐family targets (Coronaviridae, Flaviviridae, Orthomyxoviridae, Poxviridae, Arenaviridae, and Filoviridae). Bubble size corresponds to the extent of preclinical or clinical maturity (larger bubbles = more advanced clinical stage), while color intensity reflects translational readiness (Preclinical → Phase I → Phase II → Phase III). Together, the panels illustrate the shift from virus‐targeted pharmacology toward nanoenabled, cross‐family therapeutic strategies driving next‐generation pandemic preparedness.

BSAs are distinguished by their ability to target multiple viruses across different families, thus providing a versatile tool for fighting viral diseases. Unlike traditional antiviral drugs, which are typically designed to inhibit specific viral enzymes and therefore possess a narrow scope of effectiveness, BSAs offer the potential to combat a wide range of viruses—including those that have not yet emerged as significant threats (**Figure** **1**a,b). This broad targeting capability is particularly critical in pandemic situations, where the rapid spread of a novel virus leaves limited time for developing pathogen‐specific treatments.^[^ ^8^, ^9^ ^]^ The COVID‐19 pandemic has underscored the importance of such a versatile approach, revealing the limitations of existing antiviral strategies and the need for more robust and wide‐ranging solutions. In response, significant initiatives have been launched to advance the discovery and development of BSAs. Leading these efforts are organizations such as the NIH and the Bill and Melinda Gates Foundation, which have prioritized the development of BSAs as a critical component of global pandemic preparedness.^[^ ^10^ ^]^ These initiatives target the viral families most likely to cause future pandemics, ensuring that the world is better equipped to respond to the next viral threat.

Historically, the development of antiviral drugs has been hampered by the narrow focus on these treatments, a one‐drug‐one‐target approach. Many existing antivirals target specific viruses, limiting their utility in the face of emerging viral threats.^[^ ^11^ ^]^ This is in stark contrast to the broad‐spectrum action of antibiotics, such as penicillin, which can target a wide range of bacterial infections. The challenge is to replicate this broad‐spectrum approach in the field of antiviral drugs to create treatments that can be rapidly adapted and deployed in response to new viral outbreaks.

The focus on BSAs is a paradigm shift in antiviral drug development, emphasizing preparedness and rapid response. By advancing BSAs to early‐stage clinical trials, researchers and policymakers can create a stockpile of antiviral drugs that can be quickly mobilized in a new pandemic. This strategy not only aims to mitigate the impact of future viral outbreaks but also to prevent them from escalating into global health crises. Therefore, developing BSAs is a critical priority to ensure humanity is better equipped to handle future viral threats, highlighting the crucial need for innovation and investment in this area.

Niclosamide (NIC), which has shown potential against multiple viruses according to the previous in vitro studies, can play a pivotal role in this endeavor.^[^ ^12^ ^]^ Unlike traditional antivirals with a narrow scope of effectiveness, BSAs, such as NIC, offer a versatile and timely solution that could save countless lives by preventing or mitigating outbreaks before escalating into full‐blown pandemics. This realization has spurred an urgent and global call for action, emphasizing the need to develop BSAs that can respond immediately to future pandemics and viral threats, such as dengue and Mpox, to ensure a more secure and a well‐prepared world.

**Figure 2:**
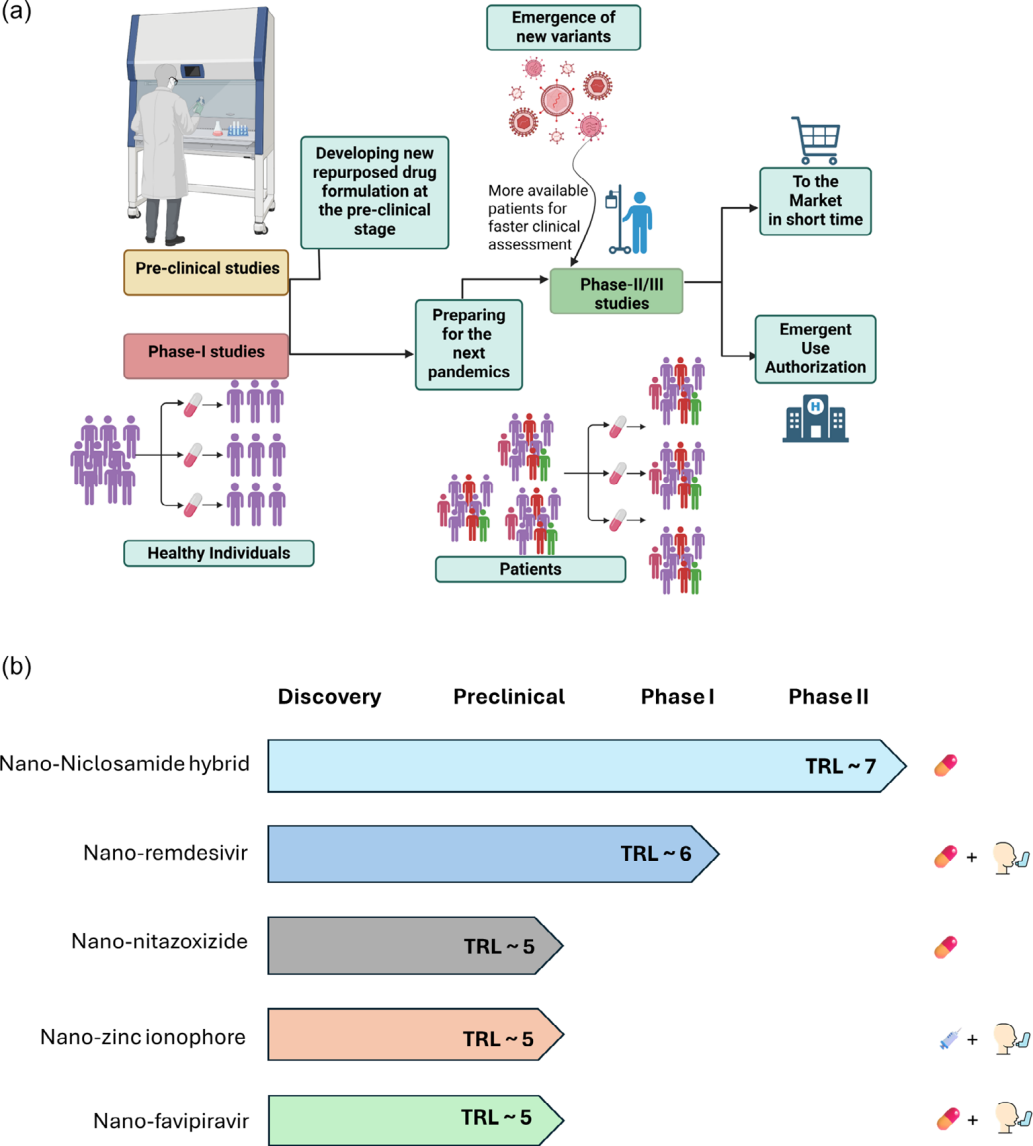
Development pipeline and TRL framework for nanoengineered BSAs. a) Strategic workflow illustrating how nanoengineered repurposed drug formulations can accelerate antiviral development and pandemic preparedness. Starting from preclinical optimization, promising candidates advance through early safety (phase I) and efficacy (phase II/III) evaluation, allowing rapid clinical translation when new viral variants emerge. This streamlined route supports emergency use authorization and shortens the time to market for effective broad‐spectrum therapeutics. b) Comparative translational readiness landscape of representative nano‐BSAs: NIC–MgO–HPMC (inorganic‐organic nanohybrid), nano‐NTZ (polymeric), nano‐favipiravir (lipid), nano‐remdesivir (lipid), and nano‐zinc‐ionophore (lipid). Horizontal bars indicate progression across discovery to approval stages, with colors denoting formulation type and icons representing delivery routes (oral = pill, inhalation = inhaler device symbol, IV = syringe). *Note:* Technology Readiness Levels (TRLs) were assigned according to the European Commission Horizon 2020 Annex G and biomedical adaptations described by van Wezel et al., Nanomedicine: NBM (2021). For nanomedicine, TRL 4–5 denotes preclinical validation, TRL 6 indicates first‐in‐human safety (phase I), TRL 7 corresponds to phase II efficacy, and TRL 8–9 reflects pivotal clinical or approved status. As of late 2025, only nano‐NIC (NIC–MgO–HPMC) has reached TRL 7, supported by phase II COVID‐19 clinical data (KCT0007307). All other nano‐BSAs remain at preclinical or early translational stages.

The COVID‐19 pandemic highlighted the need for rapid‐response development pipelines (**Figure** **2**a,b) that can effectively combat emerging viral threats, showcasing the limitations of traditional drug development timelines. Developing and establishing preclinical or early‐phase clinical data for versatile, BSAs such as nanoengineered NIC could offer a strategic advantage for pandemic preparedness and response. Nanoengineered NIC, with its enhanced solubility and bioavailability, presents itself as an ideal candidate for such a framework. Preclinical studies, or phase 1 clinical trials conducted during nonpandemic periods, can provide initial safety and efficacy data that may later be rapidly leveraged during an outbreak.

This strategic approach allows for expedited advancement into later clinical phases, such as phase 2 or 3 trials, when a new viral threat emerges. The benefit of this model is a streamlined clinical development process: as soon as a novel virus poses a public health risk, nanoengineered NIC could quickly move to larger patient trials, thus accelerating the pathway to emergency use authorization and eventual full approval. By maintaining a stockpile of phase 1 data on BSA candidates, regulatory bodies can expedite phase 2 or 3 approvals, facilitating faster distribution and marketing. Such a strategy offers an innovative solution for responding to viral threats with greater agility, ensuring accessible and pre‐tested antiviral options that enhance global health security in the face of future pandemics.

To realize this fast‐response model, formulation science plays a pivotal role. Beyond pharmacological repurposing, nanotechnology has emerged as a transformative enabler for BSAs. By tailoring particle size, surface charge, and hybrid interfaces, nanoengineering enhances drug solubility, endosomal escape, and intracellular bioavailability—key determinants of antiviral efficacy. This review therefore emphasizes nanoscale design principles that underpin the improved performance of BSA candidates such as NIC, remdesivir, and favipiravir.

In parallel with these scientific advances, global policy platforms have increasingly recognized the need for adaptable therapeutic technologies that can be rapidly mobilized during future outbreaks. Recent G7 Academies’ Statements, G7 Science and Health Ministers’ Meetings, and related multilateral dialogues highlight a clear consensus: broad‐spectrum antivirals must be supported not only by molecular innovation but also by scalable, equitable, and environmentally sustainable delivery systems. These high‐level discussions underscore several converging priorities. First, the repurposing of existing FDA‐approved drugs with well‐established safety profiles—such as niclosamide—remains a central pillar of pandemic readiness. Second, nanomedicine is viewed as a critical accelerator capable of overcoming the physicochemical limitations that have historically constrained such drugs. By improving solubility, bioavailability, and targeted delivery, nanoengineered niclosamide aligns directly with the G7's call for rapidly deployable, multi‐pathogen therapeutic platforms. Moreover, G7 science‐policy forums emphasize that preparedness must extend beyond innovation to include equitable access, technology transfer, and regulatory harmonization, particularly for low‐ and middle‐income countries (LMICs). Nano‐niclosamide offers practical advantages within this framework: its production can be cost‐effective, scalable, and compatible with green chemistry principles, supporting both global health equity and environmental sustainability. These priorities are further reinforced by the One Health perspective, which stresses integrated stewardship of human, animal, and environmental health. High‐efficiency nanoformulations requiring lower doses can help reduce pharmaceutical waste and ecological burden, contributing to greener pandemic responses. Taken together, the scientific and policy landscapes converge on a shared message: the integration of nanotechnology‐enhanced therapeutics such as nano‐niclosamide into global pandemic preparedness strategies is both scientifically justified and strategically necessary. This alignment provides not only a mechanistic rationale for advancing BSA nanoplatforms but also a forward‐looking policy framework to support their global adoption.

## 2 Need for BSA Development and Associated Efforts

Reflecting the inadequate response to COVID‐19, there has been a strong call for the development of BSAs to better prepare for future pandemics. Ahead of the G7 summit held in Germany (June 2022), the national science academies of G7 countries issued a statement recommending preparedness for the next pandemic.^[^ ^13^ ^]^ The statement, titled “Antiviral Drugs: Increasing Preparedness for the Next Pandemic,” was jointly signed by the heads of the US National Academy of Sciences, Royal Society of the UK, French Academy of Sciences, German National Academy of Sciences, Royal Society of Canada, National Academy of Sciences of Italy, and the Science Council of Japan. It emphasized that unlike existing antivirals, which are only effective against individual viruses, BSAs must be developed to enable preparedness and immediate response to pandemics, highlighting the need for antivirals effective against entire viral families or even broader ranges of viruses.

Several organizations have decided to support the development of BSAs with efficacy against viral families likely to cause a pandemic. On May 18, 2022, the US NIH identified seven viral families, including Coronaviruses, Paramyxoviruses, and Bunyaviruses, aiming to develop antivirals up to the preclinical stage. To achieve this, nine US universities and research institutions were allocated $577 million in support. Additionally, the Bill & Melinda Gates Foundation, in partnership with the Novo Nordisk Foundation and Open Philanthropy, launched a new initiative, the Pandemic Antiviral Discovery (PAD), to accelerate the discovery and early development of antivirals, committing up to $90 million in initial funding.^[^ ^14^ ^]^

## 3 BSAs and Associated Challenges

The structural differences among viruses make them difficult targets for drug development. Some of the major challenges associated with BSAs are listed in **Table** **1**. Nevertheless, several antiviral agents are effective against different viral species.^[^ ^15^ ^]^

**Table 1:** Major challenges associated with present day BSAs.

Challenge	Description	Ref.	Potential Solution
Poor pharmacokinetics	Antivirals like NIC often face solubility and bioavailability issues, reducing efficacy.	[141]	Use nanoengineering to enhance solubility, stability, and targeted delivery.
Rapid viral mutation	Frequent mutations, especially in RNA viruses, lead to resistance against existing drugs.	[142, 143, 144, 145, 146, 147, 148, 149, 150, 151, 152]	Broad‐spectrum agents that act on host factors or viral replication mechanisms may reduce resistance risk.
Safety and toxicity concerns	High doses needed for efficacy may increase side effects and toxicity.	[153, 154, 155, 156, 157, 158, 159]	Use nanoengineering to enhance delivery to targeted cells, allowing for lower effective doses and reduced systemic toxicity.
Accessibility in resource‐limited areas	High drug costs limit availability in low‐income regions.	[160, 161, 162]	Develop cost‐effective formulations and manufacturing processes for affordability and widespread use in all regions.
Regulatory and approval hurdles	Extensive testing requirements delay emergency use approvals for new broad‐spectrum drugs.	[163, 164, 165]	Conduct preclinical and early‐phase trials to build foundational data for accelerated testing in response to new viral outbreaks.
High development cost	BSA research and development is expensive and time‐consuming.	[166]	Reposition existing drugs like NIC with known safety profiles to reduce costs and expedite approval processes.

The development of BSAs is challenged by the fundamental biological diversity among viruses, particularly between RNA and DNA viruses. For RNA viruses, the RNA‐dependent RNA polymerase (RdRp) serves as a central enzymatic target because it is highly conserved and essential for viral genome replication.^[^ ^1^ ^]^ Many BSAs—including favipiravir,^[^ ^16^ ^]^ remdesivir,^[^ ^17^ ^]^ and ribavirin^[^ ^18^ ^]^—achieve efficacy by inhibiting RdRp or inducing mutagenesis during RNA synthesis.

In contrast, DNA viruses rely on DNA‐dependent DNA polymerases (DdDp) for genome replication, and inhibitors of these enzymes—such as brincidofovir, an orally bioavailable lipid conjugate of cidofovir—represent BSA agents active primarily against DNA virus families including Poxviridae, Adenoviridae, and Herpesviridae.^[^ ^2^, ^19^ ^]^ Although brincidofovir mainly targets DNA viruses, some studies have reported indirect or host‐mediated effects that may modestly influence the replication of certain RNA viruses such as Ebola (EBOV),^[^ ^2^ ^]^ Marburg virus, and coronaviruses^[^ ^3^ ^]^ (including MERS‐CoV and SARS‐CoV‐2),^[^ ^3^ ^]^ and Venezuelan equine encephalitis virus (VEEV)^[^ ^4^ ^]^ and Chikungunya virus (CHIKV).^[^ ^5^ ^]^ However, these effects are not due to direct RdRp inhibition. Notably, brincidofovir use has been associated with dose‐dependent hepatotoxicity, as observed in patients treated for Mpox virus infections.^[^ ^20^ ^]^ Favipiravir (T‐705), a nucleoside analog that induces lethal mutagenesis through RNA misincorporation in its active form,^[^ ^21^ ^]^ has demonstrated in vitro antiviral efficacy against Influenza A virus (IAV; Orthomyxoviridae family),^[^ ^22^ ^]^ VEEV (Togaviridae family), SARS‐CoV‐2 (Coronaviridae family), and EBOV (Filoviridae family).^[^ ^23^ ^]^ Although approved in China and India for COVID‐19, prospective randomized studies on patients with COVID‐19 have failed to demonstrate its clinical benefits. Similarly, while favipiravir prevented death in EBOV‐infected mice, phase 2 clinical trials did not show a clinical benefit despite reducing the viral load in patients. In contrast, in a clinical trial involving patients with severe fever and thrombocytopenia syndrome (SFTS), favipiravir increased viral clearance and reduced mortality.^[^ ^24^ ^]^ Recent nanoformulations using PLGA and lipid nanoparticles have improved favipiravir's permeability and intracellular retention, enhancing its RdRp‐targeting efficiency and reducing required dosage.^[^ ^25^, ^26^ ^]^

Chloroquine and hydroxychloroquine have weak to moderate BSA activity linked to their mechanism of increasing the pH of the endosomes used by viruses as an entryway to infect cells.^[^ ^27^ ^]^ This affects the activity of pH‐dependent lysosomal/endosomal proteases, potentially inhibiting the ability of the virus to infect cells.^[^ ^28^ ^]^ Recently, the nanoencapsulation of hydroxychloroquine in polymeric micelles^[^ ^29^ ^]^ modulates endosomal pH locally and reduces cardiotoxicity, reflecting the safety advantages conferred by nanoscale delivery systems.

For COVID‐19, cell experiments have shown that the EC_50_ values of chloroquine and hydroxychloroquine against SARS‐CoV‐2 range from 0.72 to 17.31 μM.^[^ ^30^, ^31^ ^]^ In another study, 26 patients hospitalized with COVID‐19 were treated with hydroxychloroquine, and by day 6, fewer patients in the treatment group tested positive than the control group.^[^ ^32^ ^]^

On March 28, 2020, the US Food and Drug Administration (FDA) approved hydroxychloroquine for an unapproved indication (COVID‐19). However, the FDA later revoked this authorization, citing the risk of heart rhythm problems associated with hydroxychloroquine.^[^ ^33^, ^34^, ^35^, ^36^ ^]^

It was found that nanoliposomal and lipid–polymer hybrid systems for remdesivir have superior pulmonary delivery, sustained plasma levels, and reduced hepatic metabolism, showcasing nanoscale control of drug release and biodistribution.^[^ ^37^ ^]^ Research on remdesivir began at Gilead Sciences with nucleoside and nucleotide analogs targeting the RdRp of the hepatitis C virus (HCV). However, an analog (GS‐6620) chosen as an HCV treatment candidate was discontinued during phase 1 clinical trials due to variable issues.^[^ ^38^ ^]^ However, Gilead continued research on nucleoside and nucleotide analogs and developed GS‐5734 (remdesivir), a prodrug of the analog GS‐441524, with improved stability in the blood.^[^ ^39^ ^]^

An EBOV outbreak occurred in early 2014 and rapidly spread across West Africa, against which Gilead pursued remdesivir as a treatment option. In in vivo studies, remdesivir demonstrated limited oral bioavailability due to high liver clearance, especially in nonhuman primates, leading to the development of remdesivir with a focus on intravenous (IV) administration.^[^ ^40^, ^41^ ^]^

However, in a randomized controlled trial conducted in the Democratic Republic of Congo involving patients infected with EBOV (NCT02818582), remdesivir treatment was inferior to antibody‐based therapy in terms of mortality, leading to the termination of the remdesivir intervention arm.^[^ ^42^ ^]^

The clinical safety data generated from the Ebola trial supported the entry of remdesivir into clinical trials for COVID‐19. On January 20, 2020, officials in the US confirmed the first case of COVID‐19, and the patient fully recovered following treatment with remdesivir.^[^ ^43^ ^]^ On February 6, 2020, the first clinical study involving a 10‐day course of IV remdesivir in hospitalized COVID‐19 patients was conducted in Hubei Province, China.^[^ ^44^ ^]^ The authors failed to demonstrate a significant improvement in overall mortality or time to recovery, with the median duration of symptoms before enrollment being 10 days. However, in patients with symptoms lasting <10 days, the time to clinical improvement was shorter than that in the placebo group (18 vs. 23 days). The trial was terminated locally due to a sharp drop in serious COVID‐19 cases.

Later, the multicenter ACTT‐1 trial (Adaptive COVID‐19 Treatment Trial‐1), initiated on February 21, 2020, showed that remdesivir reduced the recovery time from 15 to 10 days (*p* < 0.001) and shortened hospital stay. Mortality rates were lower on day 14 (6.7% vs. 11.9%) but not significantly by day 29 (11.4% vs. 15.2%).^[^ ^45^ ^]^ In March 2020, the WHO‐sponsored SOLIDARITY trial, which involved over 11 000 patients from 30 countries, found no significant difference in in‐hospital mortality between the remdesivir arm and standard care (12.5% vs. 12.7%; *p* = 0.50)^[^ ^46^ ^]^ Although there was controversy over the drug's effectiveness based on these clinical trial results,^[^ ^47^ ^]^ on May 1, 2020, the FDA granted emergency use authorization for remdesivir;^[^ ^48^ ^]^ in July, the EMA granted Conditional Marketing Approval, followed by full FDA approval in October 2020 under the name Veklury.^[^ ^49^ ^]^

**Figure 3:**
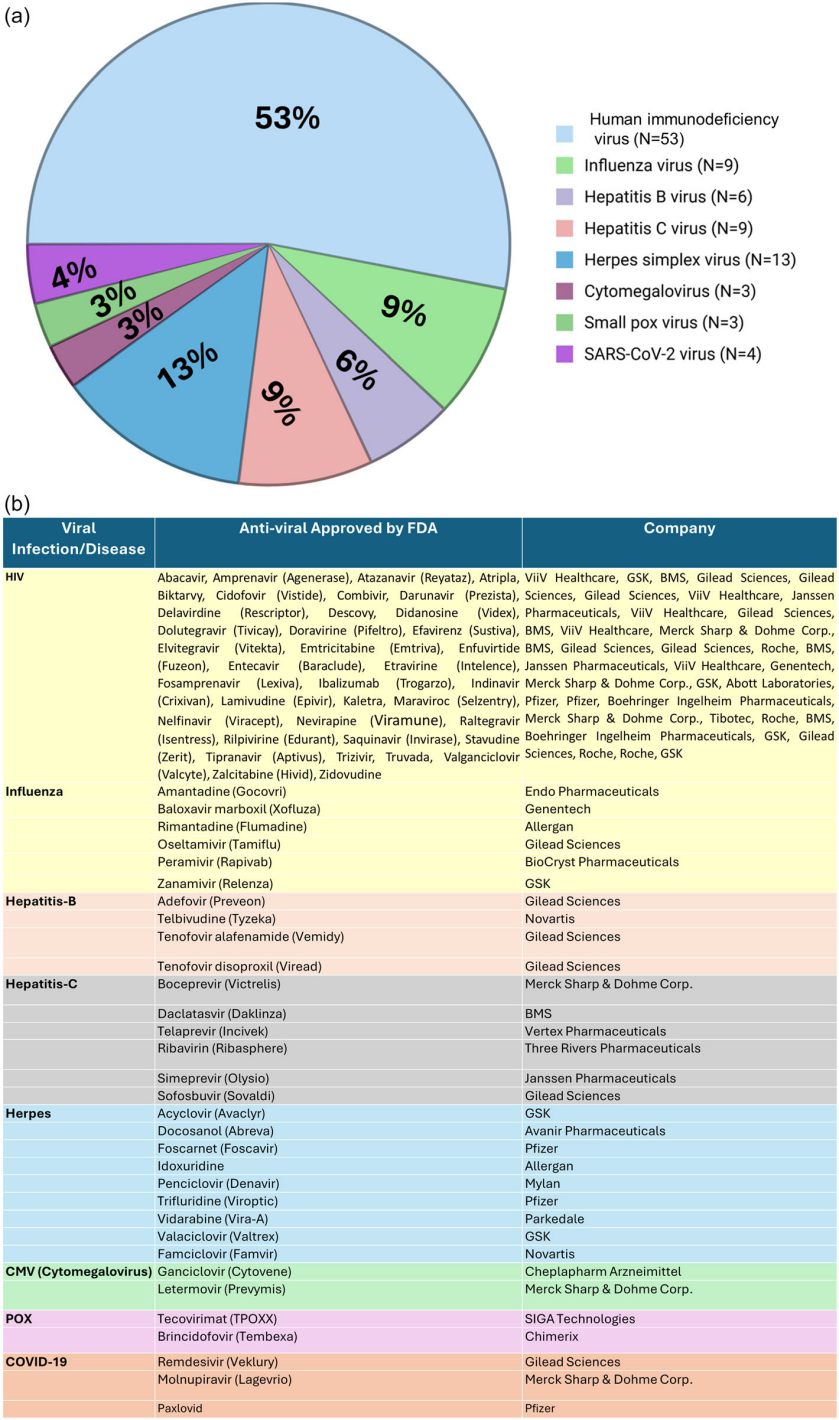
Overview of FDA‐approved antiviral drugs and their distribution across viral infections. The upper pie chart a) illustrates the proportional distribution (by number of drugs) of FDA‐approved antivirals targeting various viral pathogens. Human immunodeficiency virus (HIV) dominates with 53% of the total, followed by herpes simplex virus (13%), influenza (9%), and hepatitis B and C viruses (9% each). Cytomegalovirus (CMV), smallpox virus, and SARS‐CoV‐2 have fewer approved options. The lower table b) categorizes FDA‐approved antivirals based on the viral infection or disease they treat, alongside the commercial name and pharmaceutical company responsible for development. This visual underscore both the disparity in therapeutic focus and the commercial landscape of antiviral drug development.

Molnupiravir, another nucleoside analog that induces lethal mutagenesis,^[^ ^50^ ^]^ has demonstrated efficacy against EBOV, IAV, and SARS‐CoV‐2 in vivo. In clinical trials for mild‐to‐moderate COVID‐19, molnupiravir enhanced viral clearance and reduced mortality, leading to emergency authorization; however, its mutagenic potential remains concerning.^[^ ^51^, ^52^, ^53^, ^54^ ^]^ Current major FDA‐approved antivirals in the market from various manufacturers have been listed in **Figure** **3**.

**Figure 4:**
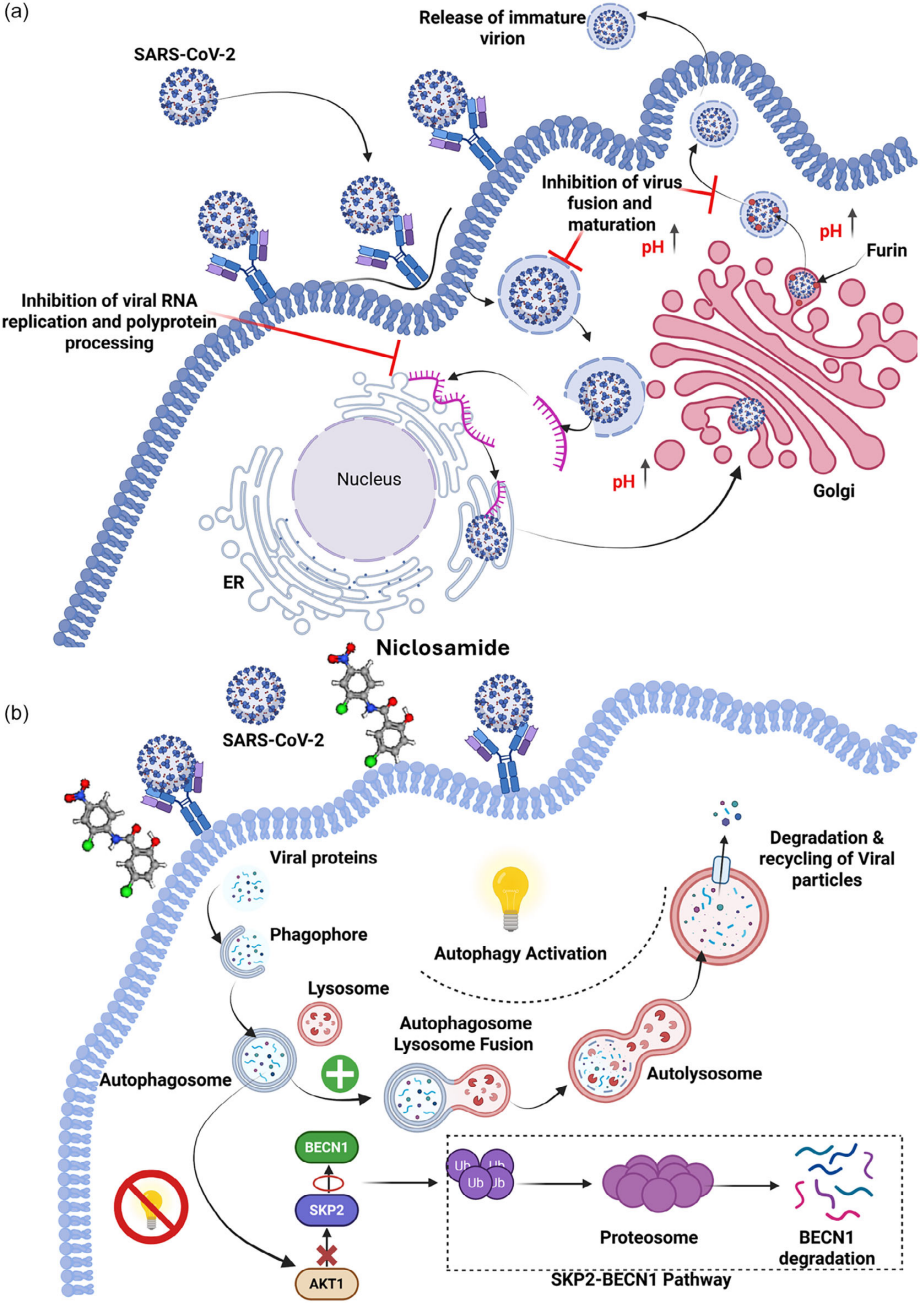
Mechanistic insights into NIC‐mediated antiviral activity against SARS‐CoV‐2. a) Schematic representation of the intracellular life cycle of SARS‐CoV‐2 and the potential inhibitory mechanisms of NIC. The drug interferes with multiple stages of viral infection, including (i) inhibition of viral RNA replication and polyprotein processing in the endoplasmic reticulum (ER); (ii) disruption of virus‐cell membrane fusion and endosomal maturation due to pH modulation; and (iii) prevention of proper virion maturation in the Golgi apparatus via furin inhibition, ultimately leading to the release of immature virions. b) Autophagy‐mediated antiviral response enhanced by NIC. The drug inhibits the SKP2–BECLIN1 (BECN1) signaling axis, leading to activation of autophagy. This promotes the formation of autophagosomes, their fusion with lysosomes to form autolysosomes, and subsequent degradation and recycling of viral components. Downregulation of SKP2 prevents BECN1 degradation, thereby facilitating sustained autophagy and viral clearance.

NIC, originally developed as an anthelmintic, has shown promise as a host‐directed antiviral agent against SARS‐CoV‐2 and MERS by inducing autophagy and eliminating intracellular viruses (**Figure** **4**).^[^ ^55^, ^56^ ^]^ Autophagy degrades pathogens, including viruses. NIC inhibits SKP2,^[^ ^57^ ^]^ thereby inhibiting proteasomal degradation of the autophagy regulator BECN1 and inducing autophagy. NIC has demonstrated antiviral activity against 32 virus species, including influenza, dengue, Ebola, hepatitis C, Zika viruses, and SARS‐CoV‐2,^[^ ^58^ ^]^ which shows a broader range than that of other antivirals.

The BSA activity of NIC may be, at least in part, attributed to its ability to induce autophagy along with other processes, aiding in viral degradation.^[^ ^12^ ^]^ The protonophoric activity of NIC induces endosomal neutralization, preventing viral release from the endosomes into the cytosol, thereby inhibiting viral replication. Additionally, NIC downregulates the lipids necessary for viral production^[^ ^59^ ^]^ and prevents syncytia formation involved in viral proliferation.^[^ ^60^ ^]^ The multiple antiviral mechanisms of NIC make it less likely for viruses to develop resistance in contrast to antivirals with a single antiviral mechanism.

However, the low solubility and poor bioavailability of NIC present significant drawbacks that make it difficult to maintain plasma concentrations necessary for effective antiviral action. This absorption challenge, which has persisted for more than 60 years, has hindered NICs’ ability to enter infected cells and exert their antiviral effects. Advances in drug delivery technologies have addressed these issues. Nanoengineered NIC has shown antiviral efficacy in COVID‐19 animal studies and has entered clinical trials involving patients with mild to moderate COVID‐19, confirming its antiviral effects.^[^ ^61^ ^]^

**Figure 5:**
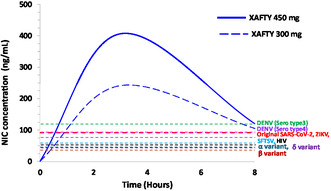
Broad spectrum characteristics of NIC toward emerging viral infections; plasma concentration‐time profiles of nanoengineered NIC (NIC) following oral administration of XAFTY 300 mg and XAFTY 450 mg in humans. The graph illustrates NIC plasma levels (ng mL^−1^) over 8 h post‐administration. The red and blue curves represent the 450 mg and 300 mg XAFTY (nanoengineered NIC hybrid), respectively. Dashed horizontal lines denote the in vitro antiviral effective concentration (EC_50_ or EC_90_) thresholds for various viruses, including 1) DENV serotype 3 (green); 2) DENV serotype 4 (magenta); 3) Original SARS‐CoV‐2 and ZIKV (red); 4) SFTSV (cyan) HIV (black); and 5) Alpha (α), Beta (β), and Delta (δ) SARS‐CoV‐2 variants (dark blue, brown, and purple, respectively). The results show that both XAFTY (which is nanoengineered NIC hybrid) achieve plasma NIC levels exceeding the EC_50_ of several high‐priority viral targets, with the 450 mg dose maintaining superior exposure across all time points. These pharmacokinetic profiles highlight the therapeutic potential of nanoengineered NIC for BSA activity and support its use in pandemic preparedness initiatives.

Nanoengineering of NIC with magnesium oxide (MgO) and hydroxypropyl methylcellulose (HPMC) to create a hydrophilic NIC–MgO–HPMC hybrid drug showed enhanced therapeutic effects in COVID‐19 patients. This innovative nanohybridization technology resulted in enhancing the NIC's intestinal permeability without altering its metabolism. The improved nanoengineered NIC showed a significant inhibitory effect against SARS‐CoV‐2 replication in a Syrian hamster model, effectively reducing lung injury associated with the virus concentration. Clinical trial results indicate that the bioavailability of nanoengineered NIC has increased approximately four times more than that of the pristine NIC, and its plasma concentration increased in a dose‐dependent manner, underscoring its potential as an effective antiviral treatment across a spectrum of viral infections (**Figure** **5**).^[^ ^61^ ^]^

## 4 Nanoengineering Strategies Driving BSA Efficacy

Recent advances in nanotechnology have transformed BSAs from pharmacological repurposing efforts into precision‐engineered nanotherapeutics. Nanoengineering enhances antiviral efficacy through physicochemical control over particle size, surface charge, composition, and release kinetics, enabling drugs to overcome biological barriers that limit free‐molecule performance. These nanoscale strategies collectively determine absorption, biodistribution, and intracellular localization—key parameters governing therapeutic success against emerging viral pathogens.

### 4.1 Particle Size and Surface Effects

Nanoparticle size is one of the most critical determinants of antiviral performance.^[^ ^62^ ^]^ Formulations in the 50–200 nm range display improved mucus penetration,^[^ ^63^ ^]^ *trans*‐epithelial transport, and cellular uptake compared with microscale suspensions. Surface charge further modulates electrostatic interactions with mucins and cell membranes:^[^ ^64^ ^]^ mildly positive or zwitterionic surfaces enhance adhesion and endocytosis without inducing cytotoxicity. For example, sub‐100 nm polymeric or lipid nanoparticles carrying antiviral payloads can efficiently traverse pulmonary mucus or intestinal barriers, achieving rapid systemic absorption. These properties are particularly advantageous for respiratory viruses, where deep‐lung deposition and cellular internalization are essential for therapeutic activity.

### 4.2 Hybrid Composition and Inorganic–Polymer Interfaces

Hybrid nanocarriers integrate inorganic cores with polymeric or biopolymeric shells to combine structural stability and controlled dissolution. The MgO–HPMC hybrid of NIC (NIC–MgO–HPMC) exemplifies this concept.^[^ ^65^ ^]^ MgO nanoparticles increase the local micro‐pH, improving the solubility of weakly acidic NIC, while the HPMC matrix provides hydrophilicity and mucoadhesion. This synergistic architecture prevents drug crystallization, shields NIC from acidic degradation, and promotes gradual release in intestinal fluid. Similar hybrid designs—such as silica‐polymer or layered double hydroxide (LDH)‐biopolymer composites—offer tunable surface chemistry and drug–carrier interactions, enabling broad adaptation to different antiviral chemotypes.^[^ ^66^ ^]^

### 4.3 Controlled Release and Endosomal Escape

Nanoencapsulation facilitates controlled, sustained release profiles that maintain plasma concentrations within the therapeutic window for extended durations. Polymeric nanoparticles^[^ ^67^ ^]^ (e.g., PLGA and PEGylated micelles) and lipid nanocarriers (e.g., solid lipid nanoparticles and nanostructured lipid carriers) release encapsulated drugs through diffusion and matrix erosion mechanisms, reducing dosing frequency and systemic toxicity.^[^ ^68^ ^]^ Moreover, many nanocarriers promote endosomal escape—a critical step for intracellularly active antivirals. Proton‐sponge or pH‐responsive polymers buffer acidic endosomes, enabling release of the payload into the cytosol, where replication complexes of RNA viruses reside. In NIC‐based hybrids, endosomal pH neutralization additionally interferes directly with viral uncoating and replication, coupling carrier behavior with therapeutic mechanism.

### 4.4 Targeted Organ Distribution and Barrier Penetration

Nanoparticle architecture dictates tissue tropism and pharmacokinetics.^[^ ^69^ ^]^ Lipid‐based systems mimic biological membranes and accumulate efficiently in the lungs and liver—primary replication sites for coronaviruses, flaviviruses, and filoviruses. Surface PEGylation^[^ ^70^, ^71^, ^72^ ^]^ extends circulation time and enhances passive accumulation in inflamed tissues via the enhanced permeability and retention (EPR) effect. Inhalable nanoaerosols and intranasal formulations achieve localized delivery to respiratory epithelia, minimizing systemic exposure. For instance, lipid‐nanoparticle‐encapsulated remdesivir^[^ ^73^ ^]^ has shown improved pulmonary bioavailability and reduced hepatic metabolism, while polymeric favipiravir nanosuspensions prolong systemic half‐life and enhance intracellular nucleotide conversion.

### 4.5 Mechanistic Examples Across Representative BSAs

Nano‐remdesivir: Lipid‐ or polymer‐based nanosystems protect remdesivir from rapid plasma hydrolysis and first‐pass metabolism, sustaining antiviral activity within pulmonary tissue.^[^ ^73^ ^]^

Nano‐favipiravir: Encapsulation in PLGA or lipid matrices stabilizes the active nucleoside form, enhances cellular uptake, and extends pharmacodynamic duration, improving RNA‐dependent RNA polymerase inhibition.^[^ ^74^ ^]^

Nano‐NIC: MgO–HPMC hybridization enhances aqueous solubility, facilitates endosomal neutralization, and triggers autophagy via SKP2–BECN1 signaling, yielding potent multi‐pathway antiviral effects.^[^ ^65^ ^]^

Nano‐nitazoxanide: Polymer‐coated nanoparticles enable targeted interferon induction while avoiding gastrointestinal degradation, strengthening host‐directed immunity.^[^ ^75^ ^]^

Collectively, these examples demonstrate how nanoscale manipulation—of structure, interface, and release—translates into measurable improvements in antiviral potency, safety, and spectrum. The integration of physicochemical design with pharmacological repurposing thus defines a new paradigm of nanoenabled BSAs, bridging materials science, virology, and translational medicine through nanoscale design principles that exemplify the interdisciplinary vision of next‐generation antiviral research.

## 5 Comparative Landscape of Nanoengineered BSAs: Strengths, Weaknesses, and Translational Readiness

The emergence of nanotechnology as a translational accelerator has reshaped how BSAs are optimized for rapid deployment. Conventional BSAs often face pharmacokinetic and delivery bottlenecks—poor solubility, short half‐life, and off‐target toxicity—that limit their pandemic‐readiness. Nanoengineering strategies address these challenges by enhancing solubility, prolonging systemic circulation, enabling tissue‐specific accumulation, and reducing systemic exposure.^[^ ^76^, ^77^, ^78^, ^79^, ^80^, ^81^, ^82^, ^83^, ^84^, ^85^, ^86^ ^]^ Current advances can be broadly categorized into four design frameworks: (i) lipid‐based carriers (liposomes, lipid nanoparticles, and solid lipid and nanostructured lipid carriers), (ii) polymeric systems (PLGA, chitosan, HPMC, and PEG micelles), (iii) inorganic or hybrid nanocarriers (MgO, LDH, SiO_2_, and metal‐oxide composites), and (iv) biomimetic or host‐cell‐derived vesicles that fuse pharmacological precision with immunomodulation.

### 5.1 Nanoengineered NIC (NIC–MgO–HPMC)

NIC remains the most mature nanorepurposed antiviral platform. The hydrophilic MgO–HPMC hybrid (commercial code XAFTY) improved the drug's dissolution and intestinal permeability four‐fold compared with pristine NIC, maintaining plasma concentrations exceeding the in vitro EC_50_ of multiple viral targets—including dengue, Zika, Mpox, and SARS‐CoV‐2 variants.^[^ ^65^, ^76^, ^80^ ^]^ The mechanistic versatility of NIC (autophagy induction, SKP2 inhibition, endosomal pH neutralization, and lipid metabolic suppression) confers both host‐directed and virus‐directed activity, making viral resistance less likely. Human pharmacokinetic data and phase II trials in mild‐to‐moderate COVID‐19 position XAFTY at a high translational readiness level (TRL 7).^[^ ^65^ ^]^ Limitations include the need for stringent control of MgO particle size (<50 nm) to ensure reproducibility and regulatory standardization.

### 5.2 Nitazoxanide (NTZ) Nanocarriers

NTZ exhibits broad activity through interferon‐mediated host responses and inhibition of viral nucleoprotein maturation.^[^ ^87^ ^]^ Encapsulation in PLGA, chitosan, or lipid nanocarriers has increased oral bioavailability by 3–5 fold and improved pulmonary distribution. Several NTZ studies have advanced to phase III clinical evaluation for COVID‐19,^[^ ^88^ ^]^ picornavirus,^[^ ^89^ ^]^ and influenza confirming their safety and tolerability. However, NTZ's comparatively narrow chemical flexibility and rapid systemic clearance still limit sustained antiviral exposure. Combining NTZ with mucoadhesive or inhalable nanocarriers could further enhance its pandemic utility.^[^ ^89^ ^]^

### 5.3 Zinc‐Ionophore Nanodelivery (PBT2 and Hinokitiol)

Zinc‐ionophores such as PBT2 facilitate intracellular Zn^2+^ influx, which suppresses viral RNA‐dependent RNA polymerase (RdRp) activity in coronaviruses,^[^ ^90^ ^]^ picornaviruses,^[^ ^91^ ^]^ and influenza.^[^ ^92^ ^]^ Nanoencapsulation in lipid nanocapsules or silica‐based carriers enhances cellular uptake and controls zinc release kinetics, leading to synergistic inhibition when co‐formulated with other BSAs. Despite encouraging in vitro outcomes, in vivo validation remains limited, placing these formulations at an intermediate TRL 5–6. Their favorable safety margin, however, supports further development as adjunctive nanotherapies.

### 5.4 Nucleoside Analog Nanoplatforms

Remdesivir^[^ ^93^ ^]^ and its analog GS‐441524,^[^ ^94^ ^]^ as well as molnupiravir,^[^ ^50^, ^95^, ^96^, ^97^, ^98^, ^99^ ^]^ target conserved RdRp enzymes across multiple RNA virus families. Nanoengineering strategies—including lipid nanoparticles and solid dispersions—aim to overcome poor oral stability and hepatic first‐pass metabolism. Lipid‐encapsulated remdesivir has demonstrated improved pulmonary retention in rodent models,^[^ ^73^ ^]^ while nano‐molnupiravir exhibits faster gastrointestinal absorption.^[^ ^100^ ^]^ Both agents in its non‐nano froms benefit from existing regulatory approval TRL 6, though mutagenicity concerns for molnupiravir and infusion‐related toxicity for remdesivir warrant continued safety surveillance.

### 5.5 Pan‐Family Protease and Host‐Targeted Nanotherapeutics

Small‐molecule protease inhibitors such as PF‐07304814^[^ ^101^, ^102^, ^103^, ^104^, ^105^, ^106^ ^]^ and ensitrelvir,^[^ ^107^, ^108^, ^109^, ^110^, ^111^, ^112^, ^113^, ^114^, ^115^, ^116^, ^117^, ^118^ ^]^ and host‐targeted agents like camostat mesylate or zotatifin, exemplify alternative BSA directions. Nano‐camostat in polymeric micelles improves epithelial penetration and half‐life,^[^ ^119^ ^]^ while zotatifin‐loaded lipid nanoparticles suppress viral translation through eIF4A inhibition with lower systemic exposure.^[^ ^120^ ^]^ These host‐targeted nanoagents minimize resistance risk but face narrow therapeutic windows due to cytotoxicity at elevated doses. Optimizing release kinetics and selective organ targeting is essential for clinical advancement.

### 5.6 Integrative Assessment

Across these agents, nanoengineering confers three principal benefits: (1) enhanced pharmacokinetics via solubility and permeability gains, (2) dose minimization and reduced systemic toxicity, and (3) rapid translational scalability leveraging existing good manufacturing practice (GMP) frameworks. Nonetheless, heterogeneity in regulatory classification—whether considered a “drug” or a “nanomedicine”—remains a key hurdle for emergency deployment. In terms of pandemic readiness, only nano‐NIC hybrid and nano‐remdesivir are currently achieving TRL ≥ 6 with validated human safety profiles.

### 5.7 Strategic Outlook

From a systems‐preparedness viewpoint, these nano‐BSAs should not be viewed as isolated agents but as complementary components of a coordinated antiviral arsenal. Nano‐NIC provides a host‐directed, cross‐family backbone; nucleoside analogs contribute rapid‐acting, virus‐directed control; and host‐targeted protease or translation inhibitors offer resistance‐resilient adjuncts. Developing a modular BSA platform—where nanohybridization technologies (e.g., MgO hybrids, lipid NPs, polymer micelles) can be rapidly adapted to diverse actives—will enable plug‐and‐deploy therapeutic responses within weeks of outbreak detection.

Collectively, this comparative evaluation underscores that while multiple nanoenabled BSAs are progressing through the translational pipeline, nanoengineered NIC currently demonstrates the most balanced profile of mechanistic breadth, manufacturability, and clinical maturity, making it a prototype for next‐generation pandemic preparedness therapeutics.

## 6 Recommendations for Future Pandemic Preparedness

The one‐drug‐one‐target approach has long been the mainstay of antiviral development and yielded considerable success in developing antiviral agents against HIV and HCV, making AIDS a manageable chronic disease and HCV infection a curable condition. These results are remarkable, considering the relatively short history of antiviral development.

However, we encounter viruses that cause acute symptoms and pose significant threats to human life. The frequency of the outbreaks caused by these viruses has increased. Following the 1918 Spanish flu outbreak, it took 30–40 years for the Asian and Hong Kong flu outbreaks to emerge. However, since the 2000s, new viral outbreaks have emerged at intervals of 3–6 years, including SARS in 2003, H1N1 in 2009, MERS in 2012, and COVID‐19 in 2019.

Furthermore, there is an alarming phenomenon of various viruses crossing species barriers and causing outbreaks of insect‐borne viral diseases owing to climate change. On April 4, 2024, cases of bovine infection with avian influenza virus (H5N1) were first reported in Texas and Kansas, followed by cases in other states. The US health authorities believe that cattle are infected by wild birds. On April 1, one individual in Texas tested positive for H5N1 after contact with infected cattle. Scientists are concerned that this spillover event, in which H5N1 was transmitted to cattle, might lead to a viral mutation that could potentially allow human‐to‐human transmission

In 2022, as Mpox cases emerged in multiple countries, the WHO declared a Public Health Emergency of International Concern (PHEIC) on July 23, 2022.^[^ ^121^, ^122^, ^123^, ^124^, ^125^, ^126^, ^127^, ^128^, ^129^, ^130^, ^131^ ^]^ Since then, Mpox had spread to 113 countries, with ≈100 000 confirmed cases with 200 deaths (July 31, 2024). Recently, Mpox resurged in Central Africa, prompting the WHO to declare another public health emergency after 15 months. Between January and mid‐July 2024, the DRC (Democratic Republic of Congo) reported over 12 000 suspected Mpox cases^[^ ^132^ ^]^ and 447 deaths (https://www.rescue.org/kr/article/what‐mpox‐and‐how‐can‐we‐prevent‐its‐spread). The currently circulating strain of Mpox has been identified as clade 1b,^[^ ^133^ ^]^ a subvariant of clade 1 detected in Sweden,^[^ ^134^ ^]^ Pakistan,^[^ ^135^ ^]^ Philippines,^[^ ^136^ ^]^ and Thailand,^[^ ^137^ ^]^ indicating its global spread.^[^ ^138^ ^]^

Global climate change has expanded the range of vector activity. In case of dengue, ≈13 million cases and ≈8500 dengue‐related deaths have been reported globally since the beginning of 2024. Over the past two decades, the annual global incidence of dengue has also increased from 500 000 to 5.2 million cases, of which over 4.1 million are in South America. On February 8, 2024, Rio de Janeiro (Brazil) declared a public health emergency due to increasing cases of dengue fever, followed by Peru (February 27, 2024) and Puerto Rico (March 27, 2024).^[^ ^139^ ^]^

This suggests that the spread of existing viruses and the emergence of new viruses could lead to widespread outbreaks. Hence, it is crucial to develop BSAs to counteract such outbreaks by addressing the shortcomings of existing BSAs or developing new BSAs and advancing them to clinical trials to ensure their readiness for immediate use and to prevent the recurrence of the global standstill experienced during the COVID‐19 pandemic.

**Figure 6:**
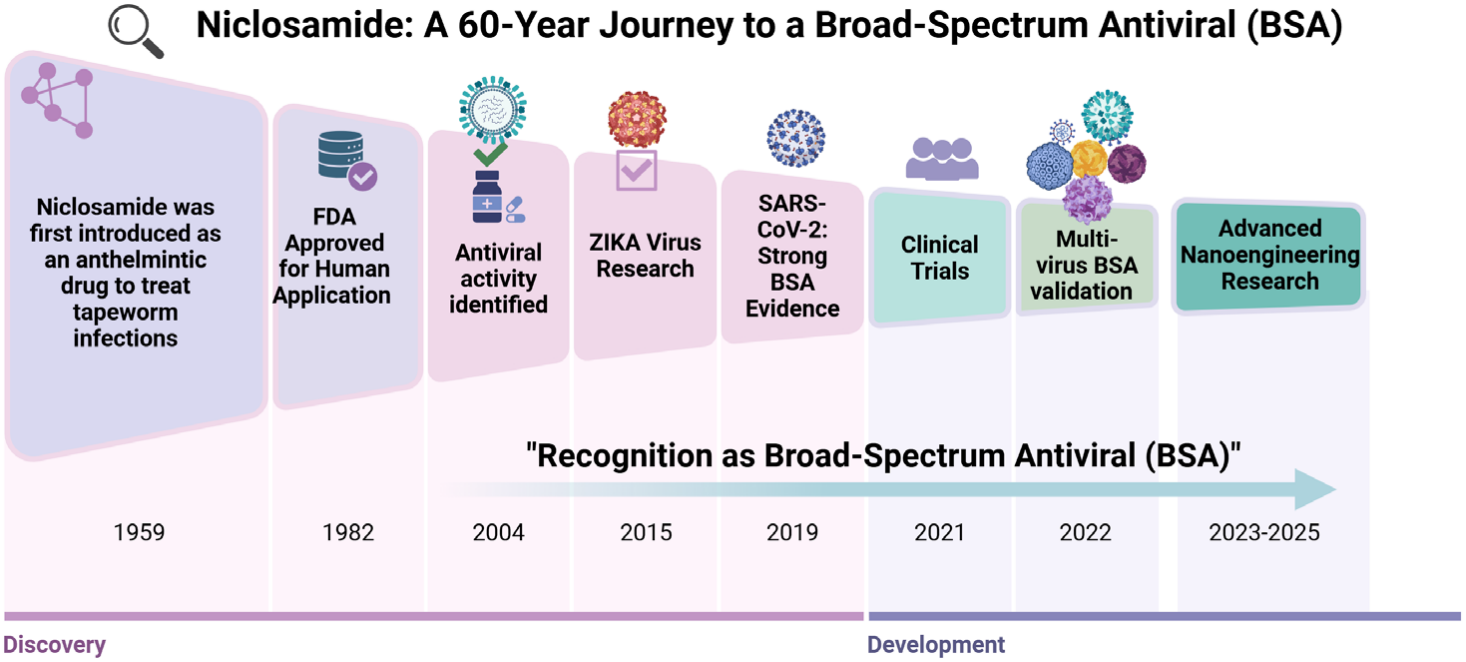
Evolution of NIC Research from Antiparasitic Agent to BSA (BSA) Candidate. This schematic illustrates the translational journey of NIC, a previously FDA‐approved antiparasitic drug, into a promising BSA candidate. The left arc outlines the historical milestones and initial discoveries, starting from its antiparasitic use and progressing through its identification of antiviral potential, including applications in Zika virus research and the COVID‐19 pandemic response. The right arc highlights recent and ongoing developments, including clinical trials for COVID‐19, expanded antiviral investigations, the creation of advanced nanoengineered hybrids, and continued efforts in optimization for BSA applications. The transition from pre‐existing approval to repurposed antiviral strategies underscores NIC's significance in global infectious disease preparedness and drug repurposing frameworks.

We believe that NIC has the most promising potential as a BSA candidate for future pandemic preparedness for several reasons. First, NIC has shown activity against 32 virus species in vitro by inducing autophagy and is expected to exhibit antiviral activity against several other virus species. Second, NIC can combat viral resistance to antivirals; its multiple antiviral mechanisms make it more difficult for viruses to develop resistance than antivirals that rely on a single mechanism. Third, NIC has a proven safety profile in humans, as approved by the FDA as an anthelmintic in 1982, and has been clinically used for over 40 years (**Figure** **6**). Yet conventional formulations have been constrained by poor solubility, instability, and limited bioavailability, underscoring the need for modern delivery strategies capable of unlocking its full pharmacological value. In this context, the recent development of CP‐COV03 (NIC–MgO–HPMC) represents a decisive advance. This nanoengineered platform combines MgO nanoparticles with HPMC through a rational safe‐by‐design approach specifically intended to enhance drug exposure while preserving long‐term safety. Importantly, a rigorous 13‐week in vivo toxicological evaluation demonstrated an outstanding safety profile: no abnormalities were observed in clinical signs, serum biochemistry, hematologic parameters, or the histopathology of major organs. These findings provide the first robust evidence that nano‐niclosamide can maintain the drug's inherent safety even under chronic exposure conditions. By establishing a clear benchmark for long‐term biocompatibility, CP‐COV03 positions nanoengineered niclosamide as a truly translatable candidate—not only for persistent viral infections such as long COVID, but also for oncology settings where extended dosing and sustained systemic exposure are essential.^[^ ^140^ ^]^ Fourth, NIC can be mass‐produced at a reasonable cost, making it globally accessible. Although the main limitation in using the pristine NIC, as a BSA for pandemic preparedness, is its low bioavailability, numerous studies have developed methods to overcome this issue. The recent clinical trial has demonstrated its improved bioavailability and safety via nanoengineering strategy.

Taken together, these advances position nanoengineered niclosamide as one of the most compelling broad‐spectrum antiviral (BSA) candidates for future pandemic preparedness. Modern nanoformulations—particularly the NIC–MgO–HPMC platform, effectively overcome the drug's historic limitations in solubility, stability, and bioavailability, enabling clinically meaningful exposure levels and sustained antiviral activity. This nanohybrid approach not only preserves NIC's inherent safety, demonstrated through decades of human use, but also extends it through rigorous long‐term toxicology studies confirming excellent biocompatibility. Importantly, nanoengineered NIC exhibits activity across multiple high‐risk viral families, including coronaviruses, orthopoxviruses, and flaviviruses such as SARS‐CoV‐2, Mpox, and Dengue, supporting its designation as a frontline BSA for rapid deployment during emerging outbreaks. By integrating cost‐effective manufacturability, broad antiviral breadth, favorable safety, and enhanced pharmacokinetics, nanoengineered NIC provides a strategically scalable therapeutic platform capable of strengthening global resilience against future pandemics.^[^ ^65^ ^]^

## 7 Other Promising BSA Platforms in Development

Beyond nanoengineered NIC, several BSA platforms are advancing through translational pipelines and collectively define the next generation of pandemic‐ready therapeutics (**Table** **2**). These include nano‐optimized zinc‐ionophores, nucleoside analogs such as nano‐favipiravir and nano‐remdesivir, and host‐directed agents like nano‐NTZ and camostat mesylate. Each uses distinct molecular mechanisms—ranging from viral polymerase or protease inhibition to modulation of host entry and translation pathways—but shares the goal of broad cross‐family antiviral coverage.

**Table 2:** Nanoengineered BSAs: mechanisms, formulation strategies, and translational readiness.

Drug/company	Mechanism of action	Target virus families	Nanoformulation type/delivery route	Clinical stage	TRL	Key translational notes	Ref/clinical trial no
Camostat mesylate (nano‐CMS)	TMPRSS2 inhibition (host protease)	*Coronaviridae*	Polymeric micelle (oral)	Preclinical	**5**	Host‐targeted; improved solubility and epithelial penetration.	[167]
Nitazoxanidea) (nano‐NTZ)	Interferon induction; viral N‐protein inhibition	*Orthomyxoviridae*, *Coronaviridae*	Polymeric nanoparticle (oral/inhalable)	Phase IIIb) (COVID‐19)	5	Improved bioavailability and mucosal delivery; suitable for host‐targeted therapies.	[168]
Favipiravir (nano‐FAV)	RdRp inhibition via nucleoside analog	*Orthomyxoviridae*, *Flaviviridae*, *Coronaviridae*	Polymeric/lipid nanosuspension (oral)	Early Phase IIb)	**5**	Enhanced stability and intracellular uptake; supports rapid oral deployment.	[169, 170]
Remdesivir (nano‐REM)/Gilead Sciences.	RdRp inhibition	*Coronaviridae*, *Filoviridae*	Lipid/liposomal (inhalation, IV)	Preclinical/early clinical	**6**	Improved lung targeting; reduced hepatic metabolism; needs phase 1 nano‐specific validation.	[171], *NCT04480333*
Zinc‐ionophore (nano‐PBT2)	Zn^2+^‐mediated RdRp suppression; host‐directed antiviral	*Coronaviridae*, *Orthomyxoviridae*, *Flaviviridae*	Lipid or silica nanocapsule (oral/inhalable)	Preclinical/early clinical	**5**	Strong host‐modulation mechanism; synergistic with nano‐NIC; next‐phase translational candidate.	[172]
Nanoformulated zotatifin, an eIF4A inhibitor	eIF4A helicase inhibition; suppresses viral protein synthesis	*Coronaviridae*, *Orthomyxoviridae*	Lipid nanoparticle (IV/oral)	Preclinical	**5**	Potent cross‐family activity; cytotoxicity limits dosage.	[173]
NIC–MgO–HPMC (nano‐NIC hybrid)/Hyundai Bioscience	Endosomal pH neutralization; SKP2 inhibition; autophagy induction	*Coronaviridae*, *Poxviridae*, *Flaviviridae, Retroviridae* *Poxviridae* *Adenoviridae* *hepeviridae* *Filoviridae* *Orthomyxoviridae* *Picornaviridae* *Herpesviridae* *Togaviridae* *Arenaviridae* *Pneumoviridae* *Rhabdoviridae*	–	Phase II/III (repurposed)	**7**	Human PK confirms >EC_50_ levels; scalable; cost‐effective nanoengineered hybrid.	[80], *KCT0007307*

Representative BSAs in clinical or translational development are summarized with emphasis on their nanoformulation type, mechanism of action, and stage of clinical maturity. Each compound is listed with its principal viral‐family targets, formulation platform, delivery route, and Technology Readiness Level (TRL 1–9). Higher TRL values denote advanced clinical or manufacturing readiness. Together, the data illustrate how nanoengineering improves pharmacokinetics, biodistribution, and efficacy across diverse antiviral scaffolds, enabling faster deployment of next‐generation pandemic countermeasures.

a) Although multiple nanoformulations of NTZ have been reported in patents (e.g., CN102526039A) and academic preclinical work, no publicly disclosed biotech or pharmaceutical company has yet advanced a nano‐NTZ formulation into a labelled commercial development pipeline as of 2025.

b) As of late 2025, no public information is available regarding phase II or phase III clinical trials involving nanoengineered formulations of NTZ or favipiravir for COVID‐19. Only their conventional (non‐nano) generic oral formulations have advanced to clinical evaluation in registered studies.

Nanoengineered hybrids have improved the bioavailability, tissue penetration, and safety of these agents, translating traditional pharmacological scaffolds into scalable therapeutic platforms. Notably, zinc‐ionophore nanocapsules enhance intracellular Zn^2+^ delivery to suppress viral polymerases, nucleoside analog nanocarriers extend plasma half‐life and reduce hepatic metabolism, and host‐targeted nanotherapeutics (e.g., polymeric camostat, lipid‐encapsulated zotatifin) minimize drug resistance while enhancing localized efficacy.

Together, these nanoenabled BSAs complement the NIC platform by demonstrating how diverse chemical classes can be engineered for rapid deployment and broad antiviral utility. Detailed comparative mechanisms, formulation strategies, and readiness levels are summarized in Table 2.

Following the comparative evaluation of nanoengineered BSAs, it becomes evident that NIC demonstrates the most comprehensive cross‐family antiviral profile to date. **Table** **3** summarizes the viral orders, families, and representative species reported to be inhibited by NIC across preclinical and translational studies. This taxonomic mapping underscores NIC's unique breadth as a BSA, extending from RNA viruses such as *SARS‐CoV‐2*, *DENV*, and *EBOV* to DNA viruses like *VACV* and *HSV*. Such cross‐lineage efficacy supports the view that nanoengineered NIC functions not as a virus‐specific antiviral but as a mechanistically versatile template for next‐generation pandemic preparedness.

**Table 3:** Representative viral families and species associated with reported or potential inhibition by NIC and nanoengineered NIC derivatives.

Order	Family	Genus	Species	Subtype	Ref.
Nidovirales	Coronaviridae	Betacoronavirus	MERS‐CoV	–	[57]
			SARS‐CoV‐1	–	[174]
			SARS‐CoV‐2	–	[175]
Ortevirales	Retroviridae	Lentivirus	Human immunodeficiency virus (HIV)	HIV‐1	[176, 177]
Chitovirales	Poxviridae	Orthopoxvirus	Vaccinia virus (VACV)	–	[178]
Rowavirales	Adenoviridae	Mastadenovirus	Human adenovirus	–	[179]
Amarillovirales	Flaviviridae	Flavivirus	Zika virus (ZIKV)	–	[180]
			Dengue virus 2 (DENV‐2)	–	[181]
			West nile virus (WNV)	–	[181]
			Yellow fever virus (YFV)	–	[181]
			Japanese encephalitis virus (JEV)	–	[181]
		Hepacivirus	Hepatitis C virus (HCV)	–	[182]
Hepelivirales	Hepeviridae	Orthohepevirus	Hepatitis E virus (HEV)	–	[183]
Mononegavirales	Filoviridae	Ebolavirus	Ebola virus (EBV)	–	[184]
Articulavirales	Orthomyxoviridae	Alphainfluenzavirus	Influenza virus (IV)	Influenza A (H1N1)	[185]
				Influenza A (H5N1)	[186]
Picornavirales	Picornaviridae	Enterovirus	Human rhinovirus (HRV)	–	[185]
			Coxsackieviruses (CV)	Coxsackie virus A21	[185]
				Coxsackie virus B4	[185]
Herpesvirales	Herpesviridae	Simplexvirus	Herpes virus (HSV)	Herpes virus 1 (HSV1)	[185]
				Herpes virus 2 (HSV2)	[187]
		Lymphocryptovirus	Epstein‐Barr virus (EBV)	–	[188]
		Rhadinovirus	Kaposi's sarcoma‐associated herpesvirus (KSHV)	–	[188]
Martellivirales	Togaviridae	Alphavirus	Chikungunya virus (CHIKV)	–	[189]
			Sindbis virus (SINV)	–	[189]
			Semliki forest virus (SFV)	–	[189]
			Ross river virus (RRV)	–	[186]
Bunyavirales	Arenaviridae	Mammarenavirus	Lassa virus (LASV)	–	[190]
	Phenuiviridae	Bandaviru*s*	Bandavirus dabieense	Severe fever with thrombocytopenia syndrome virus (SFTSV)	[191]
	Hantaviridae	–	Hantavirus (HV)	–	[192]
Mononegavirales	Pneumoviridae	Orthopneumovirus	Respiratory syncytial virus (RSV)	–	[60]
	Rhabdoviridae	Vesiculovirus	Indiana vesiculovirus (VSV)	–	[186]
		Lyssavirus	Rabies virus (RABV)	–	[186]

This table summarizes viral taxa for which NIC has demonstrated direct or mechanistically inferred antiviral activity in vitro or in vivo. In several cases, nanoengineered hybrid (e.g., NIC–MgO–HPMC and related hybrids) are proposed to enhance these effects through improved solubility, stability, and tissue targeting, though comprehensive validation remains under investigation. The listed viruses encompass both RNA and DNA families, reflecting the broad mechanistic potential of NIC as a prototype scaffold for nanoenabled BSA development.

## 8 Future Outlook: Environmental and Health System Sustainability Through Nanoengineered Therapeutics

The future of nanoengineered therapeutics extends far beyond managing viral outbreaks. These technologies are increasingly positioned as pivotal tools for advancing environmental sustainability, public health resilience, and global pharmaceutical equity.

### 8.1 Minimizing Ecological Impact of Pharmaceuticals

Traditional antiviral drugs typically demand high systemic doses to reach therapeutic thresholds, often resulting in significant pharmaceutical waste and ecotoxicological concerns. Such practices not only increase manufacturing burdens but also contribute to the accumulation of active pharmaceutical ingredients (APIs) in waterways, soil, and food chains, posing risks to aquatic and terrestrial ecosystems. Nanoengineering resolves these challenges by enabling targeted delivery and controlled release, which improves therapeutic efficiency while minimizing off‐target accumulation and environmental release. This precision allows for dose sparing, reducing the quantity of raw materials needed and the load on wastewater treatment systems.

### 8.2 Green Nanomedicine and Circular Innovation

Recent advancements highlight a move toward biocompatible and biodegradable nanocarriers, such as lipid‐based systems, polysaccharide scaffolds, and polymeric micelles synthesized via green chemistry protocols. These innovations decrease dependence on petroleum‐based materials and lower the generation of toxic solvents and by‐products. For instance, the development of eco‐friendly nanoengineered NIC hybrids using aqueous or plant‐derived solvents demonstrates how antiviral therapies can align with sustainability principles. Emerging practices in life‐cycle analysis (LCA) and environmental risk assessments (ERA) are also being incorporated at early stages of nanotherapeutic design, ensuring their long‐term ecological viability.

### 8.3 Pandemic Preparedness with Reduced Environmental Footprint

Efficient and rapid containment of infectious diseases using nanoengineered antivirals may reduce reliance on mass‐scale testing, PPE, and sanitation logistics, which were major contributors to pandemic‐related pollution. By limiting viral spread through prophylactic and early‐stage interventions, nanotherapies can curb the surges in disposable medical waste, such as masks, gloves, and test kits. This not only alleviates pressure on waste management infrastructure but also supports climate‐smart healthcare goals, especially in urban and resource‐limited settings.

### 8.4 Cross‐Sectoral Impact and One Health Synergy

Nanoengineered therapeutics contribute to a One Health framework, which recognizes the interconnectedness of human, animal, and environmental health. For example, reducing pharmaceutical pollution also diminishes the risk of antiviral resistance development in animal reservoirs and supports ecosystem integrity. Moreover, the use of nanomedicine in veterinary applications, with tailored dosage and localized delivery, further complements this integrated approach.

### 8.5 Equitable Access Through Scalable Innovation

Scalable and modular platforms for nanodrug manufacturing, including 3D printing, microfluidics, and continuous flow synthesis, are driving down costs and enhancing distribution efficiency. These platforms make it feasible to decentralize drug production closer to outbreak zones, reduce cold chain dependencies, and cut down transportation‐related emissions. Ultimately, this promotes equitable global access while maintaining a lower environmental impact.

### 8.6 Policy, Education, and Regulatory Convergence

To fully realize these benefits, future strategies must integrate environmental considerations into regulatory approvals for nanoengineered antivirals. Additionally, global education campaigns can promote public awareness about the dual health‐environmental benefits of nanomedicine. Collaboration among policymakers, pharmaceutical companies, environmental agencies, and healthcare systems is crucial to embed sustainability as a core tenet of pandemic response and drug innovation.

## 9 Concluding Remarks

Nanoengineered therapeutics are redefining the landscape of antiviral preparedness. Among them, nano‐NIC (NIC–MgO–HPMC) exemplifies how nanotechnology can transform an existing, safe pharmacophore into a scalable, broad‐spectrum platform. By enhancing solubility, pharmacokinetics, and tissue targeting, nanoformulations overcome long‐standing limitations of conventional agents and extend antiviral coverage across multiple viral families.

However, the strategic importance of nano‐NIC lies not in isolation but as part of a broader ecosystem of nanoenabled BSAs—including NTZ, favipiravir, remdesivir, and zinc‐ionophores—that collectively advance the goals of rapid, cross‐family antiviral defense. The comparative readiness mapping (Figure 2b) underscores how these formulations bridge laboratory innovation and translational application, establishing a coherent roadmap toward deployable nanomedicine platforms.

As global viral threats intensify through climate‐driven vector shifts, zoonotic spillovers, and increasing human mobility, the demand for sustainable, rapidly deployable therapeutics will continue to grow. Nanoengineered antivirals meet this challenge by combining efficacy with environmental responsibility—reducing systemic toxicity, pharmaceutical waste, and manufacturing burden through low‐dose, green‐chemistry approaches.

Moving forward, progress will depend on coordinated clinical translation, regulatory harmonization, and scalable manufacturing infrastructure that can support fast activation of nano‐BSA platforms during future outbreaks. Investing in such frameworks will ensure that nano‐NIC and related nanotherapeutics evolve from reactive interventions into proactive pillars of global pandemic resilience—uniting scientific innovation, sustainability, and public health security for the decades ahead.

## Conflict of Interest

The authors declare no conflict of interest.
